# A Simple Imaging Guide for Endovascular Thrombectomy in Acute Ischemic Stroke: From Time Window to Perfusion Mismatch and Beyond

**DOI:** 10.3389/fneur.2019.00502

**Published:** 2019-05-24

**Authors:** Wengui Yu, Wei-Jian Jiang

**Affiliations:** ^1^Department of Neurology, University of California Irvine, Irvine, CA, United States; ^2^New Era Stroke Care and Research Institute, The Rocket Force General Hospital, Beijing, China

**Keywords:** acute ischemic stroke (AIS), clinical-imaging mismatch, endovascular thrombectomy (EVT), ischemic penumbra, perfusion mismatch, symptomatic intracranial hemorrhage (sICH)

## Introduction

Recent advances in medical and endovascular therapy have revolutionized stroke care. Intravenous thrombolysis (IVT) with tissue plasminogen activator (tPA) was shown to be effective for acute ischemic stroke (AIS) within 3 h of symptom onset in 1995 (1). Subsequent studies extended the time window to 4.5 h (2, 3). However, IV tPA was not very effective for stroke from large vessel occlusion (LVO) (4, 5).

Endovascular thrombectomy (EVT) was originally reported for patients with AIS from LVO in early 2000s (6–8). In 2013, 3 prospective, multi-center randomized controlled trials (RCTs), including the Interventional Management of Stroke (IMS) III (9), Mechanical Retrieval and Recanalization of Stroke Clots Using Embolectomy (MR RESCUE) (10), and Intra-Arterial vs. Systemic Thrombolysis for Acute Ischemic Stroke (SYNTHESIS EXP) (11), failed to show significant clinical benefit of EVT over standard medical therapy. No mandatory requirement for vascular imaging to screen for LVO (9, 11), nascent devices (9–11), and slow enrollment (10) may be the major limitations of these studies. However, a *post hoc* analysis of data from IMS III showed significant outcome benefit of EVT in the subgroup of patients with proven LVO (12).

In 2015, 5 RCTs independently demonstrated the safety and efficacy of EVT for AIS from LVO in the anterior circulation within 6–12 h of symptom onset (13–17). Three additional studies reported similar findings in 2016 (18–20). In early 2018, DWI or CTP Assessment with Clinical Mismatch in the Triage of Wake-Up and Late Presenting Strokes Undergoing Neurointervention (DAWN) and Endovascular Therapy Following Imaging Evaluation for Ischemic Stroke (DEFUSE 3) trials extended the time window to 16–24 h after last known well (21, 22). These studies also showed that thrombectomy during the extended time window was not associated with significant higher risk of symptomatic intracranial hemorrhage (sICH) (21, 22).

In this review, we seek to appraise various imaging modalities used in the landmark studies and to propose a simple and efficient imaging guide for EVT in the real-world practice.

### Imaging Modalities Used in Landmark RCTs

There were great variabilities in the use of imaging tools for patient selection in the recent RCTs. The key inclusion/exclusion criteria, main imaging modalities, and the thrombectomy devices used in the landmark studies are summarized in Table 1. Non-contrast CT and CTA were used to select patients with severe deficit and low infarct volume from LVO in most of the clinical trials (13, 15–20, 22). Advanced imaging tools, including CT perfusion (CTP), diffusion/perfusion MRI, and MRA, were used to identify patients with perfusion mismatch (i.e., small infarct and large ischemic penumbra) in EXTEND-IA, SWIFT PRIME, DEFUSE 3, and EXTEND-IA TNK trials (14, 17, 22, 23), or clinical-imaging mismatch (i.e., severe deficit and small infarct volume) in the DAWN trial (21). In ESCAPE trial, multiphase CTA was used to evaluate the extent of collateral circulation and patients with no or minimal collaterals were excluded from the study (15).

**Table 1:** Landmark studies of EVT for AIS from LVO in the anterior circulation.

**Study**	**Patient (*n*)**	**Key inclusion criteria**	**Key exclusion criteria**	**Main imaging modalities**	**EVT devices**
MR CLEAN (13)	233	Age ≥ 18, NIHSS ≥ 2, LVO, IVT < 4.5 h, EVT < 6 h	BP > 185/110 mmHg, coagulopathy, active or recent hemorrhage	CT, CTA, CT perfusion (68%)	Retrievable stent
EXTEND-IA (14)	35	Age ≥ 18, NIHSS≥ 6, LVO, IVT < 4.5 h, ischemic core < 70 mL, mismatch volume ≥ 10 mL, EVT < 6 h	Intracranial hemorrhage, any terminal illness	CT, CTA, CT perfusion	Solitaire device
ESCAPE (15)	165	Age ≥ 18, NIHSS ≥ 5, LVO, IVT < 4.5 h, small infarct core, EVT < 12 h	ASPECTS 0-5, no or minimal collaterals	CT, CTA	Available thrombectomy device
SWIFT PRIME (16)	98	Age 18–80, NIHSS 8–29, LVO, IVT < 4.5 h, small to moderate infarct core, EVT < 6 h	Hemorrhage, tumor or vacuities on CT or MRI, > 1/3 MCA territory or 100 ml infarct, DWI-ASPECTS ≤ 5	CT, CTA, CT perfusion	Solitaire stent retriever
REVASCAT (17)	103	Age 18–80, NIHSS ≥ 6, LVO, IVT < 4.5 h, EVT < 8 h	Large ischemic core (ASPECTS ≤ 7 on CT or 6 on DWI MRI)	CT, CTA, MRI	Solitaire stent retriever
THERAPY (18)	108	Age 18–85, NIHSS ≥ 8, LVO, ≥ 8 mm clot length	> 1/3 MCA territory infarct, cervical ICA stenosis/occlusion	CT, CTA	Penumbra
THRACE (19)	414	Age 18–80, NIHSS 10-25, LVO, IVT < 4 h, EVT < 5 h	Cervical ICA stenosis/occlusion	CT, CTA, or MRA/MRI	Stent retriever, Penumbra
PISTE (20)	65	Age ≥18, NIHSS ≥ 6, LVO, IVT < 4.5 h, EVT < 6 h	Contraindicated for IVT, > 1/3 MCA territory infarct,	CT, CTA	Stent retriever, Penumbra
DAWN (21)	107	Age ≥ 18, NIHSS ≥ 10, LVO, small infarct core (< 1/3 MCA territory), a mismatch between clinical deficit and infarct volume EVT 6–24 h	Rapid improvement in neuro status, active or recent hemorrhage, Coagulopathy	CT, CTA, MRA, CT perfusion, MR perfusion/diffusion	Trevo retriever, Solitaire, or Penumbra
DEFUSE 3 (22)	92	Age 18–85, NIHSSS ≥ 6, LVO, ischemic core < 70 ml, mismatch ratio > 1.8, mismatch volume ≥ 15 ml, or DWI volume < 25 ml EVT 6-16 h	BP > 185/110 mmHg, coagulopathy, ASPECTS score < 6 on non-contrast CT	CT perfusion 75%, MR perfusion/diffusion 25%	Trevo retriever

### CT and Alberta Stroke Program Early CT Score (ASPECTS)

**Figure 1:**
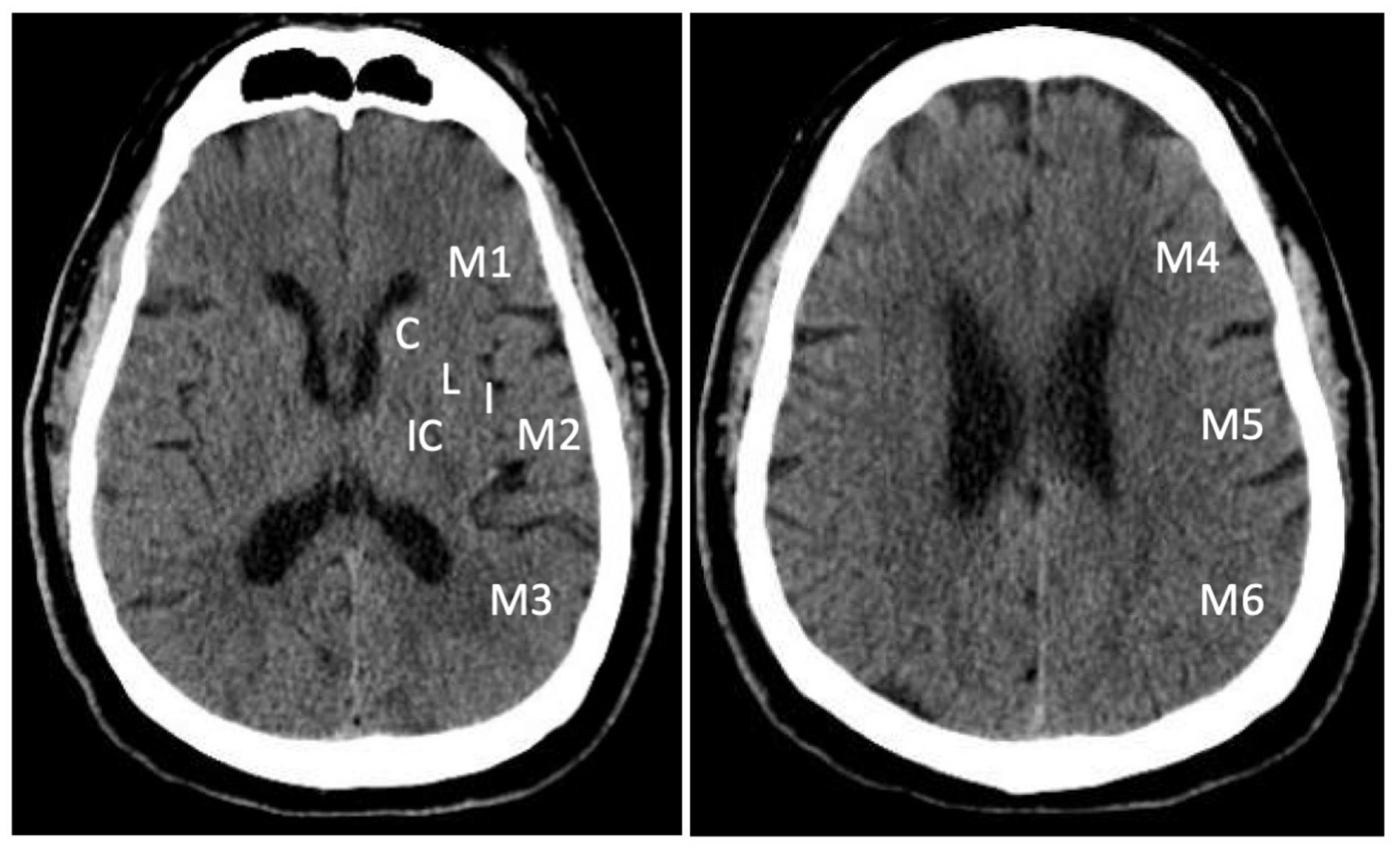
Alberta Stroke Program Early Computed Tomography Score (ASPECTS). The scoring system divides the MCA territory into 10 zones at ganglionic and supra-ganglionic levels: 6 for cortical regions (M1- M6), and 4 subcortical regions (C, caudate; L, lentiform; IC, internal capsule; and I, insular ribbon).

Non-contrast CT is widely available and can be performed within a few minutes of arrival. It is very sensitive in detecting hemorrhage (24, 25). During the first few hours of AIS, non-contrast CT is usually normal. A visible hypoattenuation on the CT is often irreversible (25). ASPECTS was developed to quantify early ischemic changes on non-contrast CT (26, 27). The scoring system divides the MCA territory into 10 zones on 2 axial CT slices at the levels of basal ganglion and the superior ganglionic margin (26). One point is subtracted for early ischemic change in each zone. A normal CT scan without any sign of ischemic change gets 10 points as shown in Figure 1. A score of 0 indicates diffuse ischemic changes in the entire MCA territory (26, 27).

ASPECTS can also be derived from CT angiographic (CTA) source image (28) or DWI image (16, 17, 29). CTA source image- and DWI-ASPECTS are more sensitive than non-contrast CT in the detection of early ischemic changes and prediction of final infarct volume (28, 29). The limitation of MRI is that it cannot be performed timely for acute stroke therapy at some medical centers.

Of note, ASPECTS score has a few limitations. First, it is limited to the anterior circulation (26). Second, it is based on anatomical structure with an unequal weighing of brain regions (30, 31). Its correlation with lesion volume is dependent on lesion location (31, 32). Last, it has poor sensitivity and inter-rater reliability for early ischemic changes (32, 33). However, the lower inter-rater reliability can be overcome by training (34).

ASPECTS score was found to be a strong predictor of clinical outcome after EVT (13, 16, 35). There was no difference in outcome between patients with ASPECTS 6-7 and 8-10 (16). A meta-analysis of the pooled data from the 5 landmark studies published in 2015 showed a clear benefit of thrombectomy in patients with ASPECT ≥ 6 (35). When the treatment effect was analyzed for the 3 ASPECTS strata of 0–5, 6–8, and 9–10, there was a strong and consistent treatment effect for both ASPECTS 6–8 and 9–10 group with an adjusted odds ratio of 2.34 (95% CI: 1.68–3.26) and 2.66 (95% CI: 1.61–4.40), respectively (35). There was no clear benefit for the 121 patients with ASPECT 0–5. These findings appear to have validated the use of ASPECTS score 6–10 as surrogate marker of small infarct volume.

### CT Angiography (CTA) for Screening for LVO

CTA of head and neck is a contrast study with high sensitivity and specificity for evaluation of cerebral vasculature and LVO (36). It also provides important information on collaterals, aortic arch, and cerebral ischemia (37–40). CTA may help interventionist to choose treatment strategy and reduce puncture-to-reperfusion time (39).

Decreased contrast enhancement on CTA source images is indicative of reduced cerebral blood volume (CBV) (34). CTA source images are more sensitive in predicting final infarct volume and outcome than non-contrast CT (28, 38, 40). Of note, slow contrast injection and quick image acquisition can lead to an overestimation of the infarct size (41).

Most landmark studies used CTA to select patients with severe deficit from LVO for EVT (13–22).

### CTA for Assessment of Collateral Circulation

Collateral circulations are highly variable among patients (38, 42). They directly affect the size of ischemic penumbra and infarct progression after LVO (37, 42–44). CTA is the most commonly used imaging modality to assess collaterals (42). Multiphase CTA is better than conventional CTA because of the ability to examine collateral flow with time resolution (38). Dynamic CTA is able to evaluate time to retrograde filling and visualize distal branches of the cerebral artery trees. Digital subtraction angiography remains the gold standard given its triphasic evaluation of arterial, capillary, and venous circulation with high temporal and spatial resolution (38, 39). The degree of leptomeningeal collaterals can be semi-quantified by comparing the retrograde pial arterial filling to the contralateral hemisphere (37, 43). A major limitation of collateral assessment on CTA is that it is a single snap shot in time of contrast and may misdiagnose adequate collaterals as poor if the image is acquired early in the arterial phase (42, 43).

Optimal collateral circulation is associated with slower infarct progression and may allow for EVT outside of the traditional time window (43, 45). A good leptomeningeal collateral flow is associated with better outcome, lower rates of sICH and mortality after EVT (42, 44, 46, 47). A large infarct core and poor collaterals were shown to be strong predictors of poor functional outcome (46, 47). Based on these findings, the ESCAPE trial excluded patients with minimal or no pial collaterals (15). Collateral assessment on CTA matched with the ASPECTS score on non-contrast CT. Minimal or no pial collaterals in >50% of MCA distribution was associated with an ASPECTS score of 5 or less (39). The DAWN and DEFUSE 3 trials demonstrated the benefit of late recanalization within 16–24 h using clinical-infarct mismatch profile indicative of good collaterals (21, 22).

Recent systemic review and meta-analysis have confirmed the favorable impact of good collateral status on functional outcome after EVT (44, 48, 49).

### Magnetic Resonance Imaging (MRI) and Magnetic Resonance Angiography (MRA)

MRI/MRA can also be used to evaluate AIS and LVO. Diffusion weighted image (DWI) is highly sensitive and specific for the detection of early ischemic changes within the first 6 h of symptom onset (50–52). Early reversible ischemia has very mild depression in apparent diffusion coefficient (ADC) due to mild reduction in cerebral blood flow (CBF) (50, 51). Timely reperfusion therapy may reverse diffusion abnormalities (52). In the absence of reperfusion, diffusion abnormalities are often irreversible (52, 53). A good stroke MR protocol should include DWI, FLAIR, and SWI (33, 54).

MRA is a good option for assessment of LVO and collateral circulation (33, 55). Time-of-flight (TOF) and contrast-enhanced (CE) MRA provide good vascular images through the neck and the Circle of Willis (55). CE MRA is performed with a rapid, short repetition time gradient echo sequence following an IV bolus of gadolinium. It is minimally invasive and offers better diagnostic accuracy than TOF-MRA in localizing LVO (55).

MRI/MRA were used as imaging tools in a few landmark studies (16, 17, 19–22).

In SWIFT PRIME and REVASCAT, 17.4% and 5.3% of patients had MRI studies for patient screening (15, 16). The DAWN and DEFUSE 3 trials used more advanced imaging tools, including diffusion/perfusion MRI, for patient selection (21, 22).

Of note, the use of MRI/MRA for patient selection has some drawbacks. It takes time to screen the patients for metallic implants and to access the scanner (33, 54, 55). The images tend to be more susceptible to patient motion. In addition, it is more difficult to monitor unstable patients in the MRI suite (33, 54).

### CT Perfusion (CTP) and Diffusion/Perfusion MRI

Acute LVO may lead to significant reduction of cerebral blood flow (CBF), resulting in a small irreversible infarct core and surrounding area of ischemic tissue that may be salvaged with prompt reperfusion (ischemic penumbra) (56, 57). Without reperfusion, the infarct core can expand and reach the size of the ischemic penumbra depending on duration of LVO and collaterals (56, 57).

CTP is a dynamic contrast-enhanced study developed for the analysis of the infarct core and ischemic penumbra per CBF, mean transition time (MTT) and cerebral blood volume (CBV) (58–62). The infarct core is defined as an area of brain tissue with more than 70% reduction in CBF compared to normal contralateral hemisphere and the ischemic penumbra is defined as an area with > 6 s of delayed arrival of contrast (39, 59–62). The ischemic penumbra is identified by a mismatch between CBF and CBV, whereas the infarct core has a matched decrease in both CBF and CBV (61–64). The mismatch between infarct core and penumbra is an indirect measurement of collateral blood flow (49).

The diffusion/perfusion MRI is very sensitive in the detection of infarct core and perfusion mismatch (51, 61, 65–70). MRI may predict clinical response to early reperfusion therapy (65–70). However, tissue at risk can be overestimated by perfusion-weighted imaging (71).

Both CTP and MR perfusion images can be obtained with high-speed CT and MR imaging systems within 10 min (22, 61). The data processing is similar. CTP or diffusion/perfusion MRI was used to assess infarct core and ischemic penumbra in EXTEND-IA, SWIFT PRIME, EXTEND IA-TNK, DAWN, and DEFUSE 3 (14, 16, 21–23).

CTP was performed in 66.8% of the patients in the MR CLEAN trial (13). It was shown that a large infarct core was associated with poor functional outcome. Both EXTEND-IA and SWIFT-PRIME used CTP to select patients with small infarct core (IQR 4–32 and 0–16 ml, respectively) for EVT (Table 1) (14, 16). Such strict selection criteria led to the highest rate of favorable outcome ever reported with EVT (60 and 71%, respectively) (14, 16). However, these studies may have excluded patients who could benefit from EVT (16, 64, 72, 73).

The DAWN trial evaluated the safety and efficacy of EVT for patients with LVO within 6–24 h of last known well (21). Approximate 60% of the patients had wake-up stroke. The key inclusion criteria were severe clinical deficit and a small infarct core on MRI or CTP. The rate of functional independence at 90 days was 49% after EVT as compared to 13% in the control group.

In DEFUSE 3 trial, CTP was performed in 73% of the patients and diffusion/perfusion MRI was done in the other 27% (22). Inclusion criteria includes an initial infarct volume < 70 ml, a ratio of ischemic penumbra to infarct core ≥ 1.8, and an absolute mismatch ≥ 15 ml. The study enrolled patients with perfusion mismatch for EVT within 6–16 h after last known well. The rate of functional independence at 90 days was significantly higher than control group (45 vs. 17%) (22).

Both DAWN and DEFUSE 3 trials demonstrated significant benefit of EVT within 16–24 h of last known well by selecting patients with clinical-imaging mismatch (i.e., severe deficit and small infarct core) per advanced imaging tools. The median NIHSS score with IQR was 17 (13–21) and 16 (10–20) while the median infarct core with IQR was 7.6 (2–18) and 9.4 (2.3–25.6) ml, respectively (Table 2) (21, 22). These results led to a paradigm shift from “time window” to “tissue window” per advanced perfusion imaging.

**Table 2:** Clinical-infarct volume mismatch as eligibility criteria for EVT in recent landmark studies.

	**Median NIHSS (IQR)**	**Median ASPECTS (IQR)**	**Median infarct core per advanced imaging-ml (IQR)^a^**	**sICH^b^ (%)**	**Favorable outcome (%)**
MR CLEAN (13)	17 (14–21)	9 (7–10)	-	7.7	33
EXTEND-IA (14)	17 (13–20)	NR	12 (4–32)	0	71
ESCAPE (15)	16 (13–20)	9 (8–10)	-	3.6	53
SWIFT PRIME (16)	17 (13–20)	9 (8–10)	6 (0–16)	1.0	60
REVASCAT (17)	17 (14–20)	7 (6–9)	-	1.9	44
THERAPY (18)	17 (14–21)	7.5 (6–9)	-	9.3	38
THRACE (19)	18 (15–21)	5–10	-	2	53
PISTE (20)	18 (6–24)	5–10	-	0	51
DAWN (21)	17 (13–21)	NR	7.6 (2.0–18.0)	6	49
DEFUSE 3(22)	16 (10–20)	8 (7–9)	9.4 (2.3–25.6)	7	45

*IQR, interquartile range; NR, not reported*.

a *Advanced imaging of perfusion CT or diffusion/perfusion MRI was used to quantify infarct core and ischemic penumbra (14, 16, 21, 22)*.

b *sICH was defined as intraparenchymal hematoma, subarachnoid hemorrhage, or intraventricular hemorrhage associated with a worsening of the NIHSS score by ≥ 4 points within 24 h (3)*.

### Patient Selection per Perfusion Imaging and Beyond

Recent studies suggested that the selection criteria per advanced perfusion imaging in Dawn and DEFUSE 3 trials may have excluded a significant proportion of patients who could benefit from EVT. In a single center study of 79 patients comparing admission infarct core per CTP and final infarct on followup CT, Boned et al. showed that CTP overestimated infarct core for more than 10 mL in 38% of the patients (72). Therefore, CTP-based patient selection may deny treatment to patients who might benefit from reperfusion therapy. In a matched case-controlled study of patients with LVO on CTA and baseline ischemic core >50 mL on CTP, EVT was associated with significantly improved functional outcome at 90 days (73). In a study of prospectively collected data, 38% of the DAWN-ineligible patients and 41% of DEFUSE 3- ineligible patients achieved functional independency at 90 days after EVT (74). In another retrospective study, 30% of DAWN and/or DEFUSE-3 ineligible patients achieved functional independence after off-label EVT (75). Two additional studies showed that EVT could benefit patients with large infarct core (DWI-ASPECTS ≤ 5 or DWI lesion > 70 mL) (76, 77). EVT was also reported to be safe and effective for patients who met all DAWN trial criteria but were treated beyond 24 h of last known well (78).

**Figure 2:**
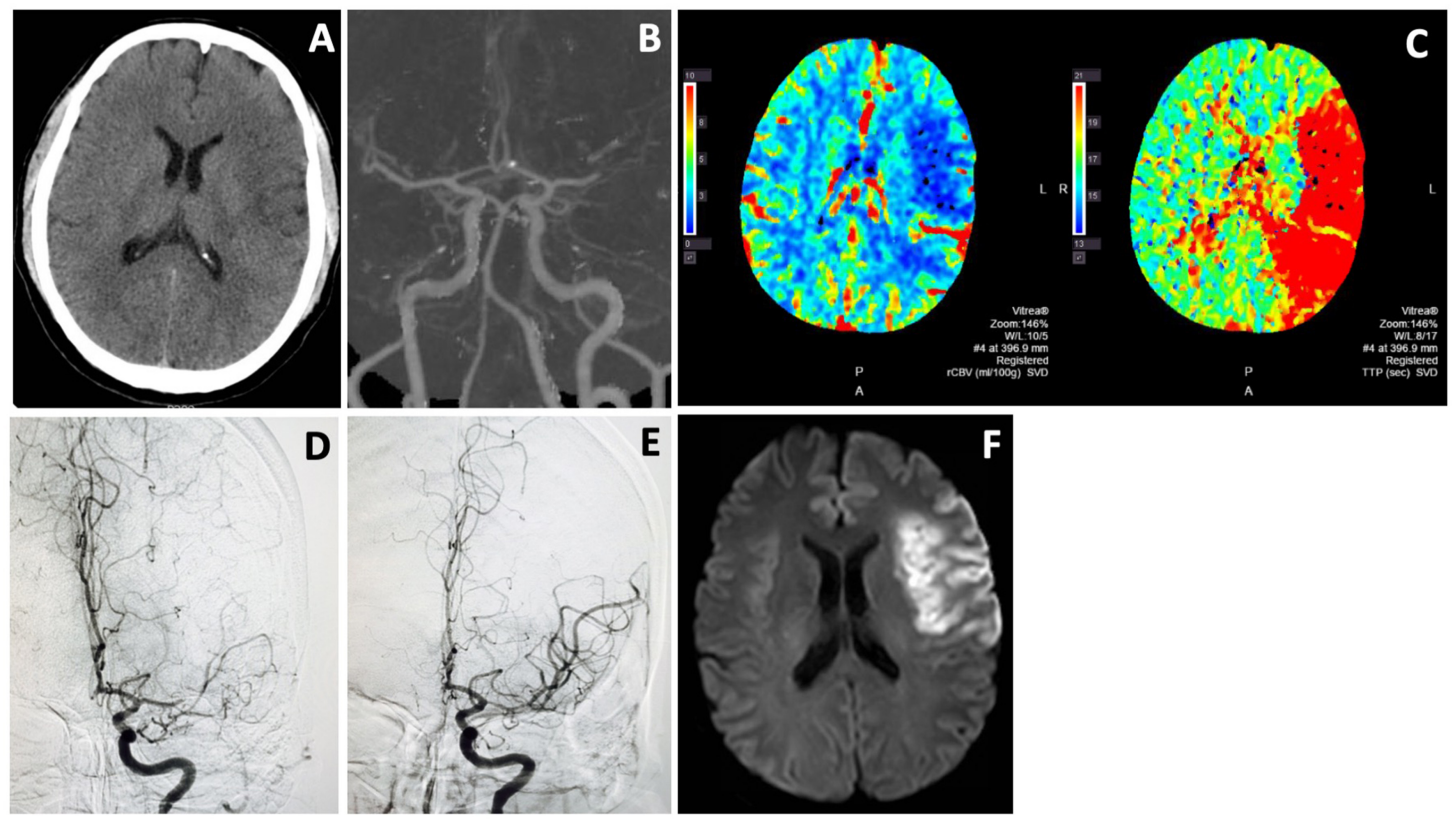
Endovascular thrombectomy for wake-up stroke. A 44 years old man woke up with right sided weakness and global aphasia. Last known well was 8 p.m. the night before. NIHSS score was 15. Non-contrast CT showed subtle left frontal hypodensity with ASPECTS of 8 **(A)**. CTA showed L MCA M1 occlusion **(B)** and CTP revealed a large ischemic penumbra **(C)**. He underwent thrombectomy with excellent MCA recanalization **(D,E)**. Follow-up MRI **(F)** showed an infarct in the left frontal region that was similar in size to the hypodense area on non-contrast CT and infarct core on CTP. He recovered well with only mild expressive aphasia at 3 month.

Figure 2 showed a typical example of EVT for wake-up stroke from middle cerebral artery occlusion. CTP or diffusion/perfusion MRI may be unnecessary in clinical practice in appropriately selected patients (13, 15–20).

### Major Complication of EVT: Symptomatic Intracranial Hemorrhage (sICH)

The periprocedural sICH is the most feared complication of EVT (13–22, 57). Early pathophysiological responses to sudden LVO are distal vasodilation to compensate for dramatic reduction in tissue perfusion and subsequent loss of vascular reactivity. Reperfusion leads to blood overflow into the dilated vasculature, resulting in hyperperfusion, cerebral edema, capillary leak, or hemorrhage (57, 79). Endothelial cell injury and impairment of the blood-brain barrier (BBB) are likely the underlying mechanism of ICH (79). The primary predictors of sICH are infarct volume (80), low CBV (70), and severely delayed CBF due to poor collaterals (81). Intensive management of high blood pressure may reduce the risk of reperfusion injury and sICH (82).

The rate of sICH from EVT ranged from 0 to 9.3% in the 10 RCTs (Table 2). That was comparable to the risk of sICH in the medical arms (13–22). Meta-analysis of data from the 5 landmark studies published in 2015 showed that the rate of sICH increased only slightly with delayed EVT (35). EVT within 16–24 h of last known well was not associated with significant higher rate of sICH (21, 22).

### Perspectives: From Time Window to Perfusion Mismatch and Beyond

Advanced perfusion imaging used in the 4 landmark studies has helped demonstrating the best treatment effect of EVT (14, 16). and extending the treatment window up to 16–24 h of last known well (21, 22). However, the median infarct core was only 12, 6, 7.6, and 9.4 ml in EXTEND-IA, SWIFT PRIME, DAWN, and DEFUSE 3 trials, respectively, (Table 2) (14, 16, 21, 22), as compared to 49.7 ml in MR CLEAN (13, 83). Therefore, the best treatment effect in the studies using advance perfusion imaging is likely the results of strict selection of patients with small infarct core for EVT (14, 16, 21, 22). There are increasing evidence to suggest the limitations of advanced imaging modalities in the real-world practice.

First, in a recent systematic review and meta-analysis of individual patient data from all recent RCTs that compared EVT with standard medical therapy, perfusion mismatch was not associated with either functional independence or functional improvement (84). Patient should not be excluded from EVT within 6 h of stroke onset purely on the basis of a large estimated ischemic core.

Second, the use of perfusion imaging for patient selection may cause delay in reperfusion therapy (22, 55). In a meta-analysis of pooled data from the 5 RCTs published in 2015, earlier treatment with EVT was associated with lower degrees of disability (84). The more recent meta-analysis showed that 30-min delay in imaging-to-reperfusion time had a similar adverse effect on functional outcome as a 10-ml increase in ischemic core volume (85). In a recent cohort study, the use of advanced modality imaging was shown to delay EVT without improvement in clinical outcomes (86).

Third, the selection criteria per advanced perfusion imaging may exclude a significant proportion of eligible patients (16, 73–77). CTP and MRI diffusion/perfusion were shown to overestimate infarct core (71, 72). A number of recent studies demonstrated that thrombectomy may benefit DAWN and/or DEFUSE-3 ineligible patients (73–77).

Last, perfusion imaging capability is not readily available, in particular, in developing regions. A significant proportion of eligible patients world-wide would be deprived from the proven therapy if perfusion imaging criteria be strictly adhered to in clinical practice.

When designing clinical trials, it makes sense to use advanced imaging tools for patient selection in order to achieve the best treatment effect in small sample size studies. Since EVT has been independently proven effective by 10 RCTs (13–22), it is imperative to provide the therapy to all eligible patients in the fastest puncture-to-reperfusion time.

Of the 10 RCTs that independently demonstrated the powerful efficacy of EVT, 8 validated the use of ASPECTS score for the assessment of early infarct (13, 15, 17–20, 22). As shown in Table 2, clinical-imaging mismatch (i.e., high NIHSS and ASPECTS) is clearly a good indication for EVT in the real-world practice.

### Proposed Simple Imaging Guide for EVT

The ideal imaging guide for decision-making for EVT should be widely available, quick to perform and interpret, and sensitive for the detection of early infarct, LVO and collaterals (13–22, 39).

NIHSS is a good surrogate marker for clinical deficit (87, 88) and ASPECTS has been validated for the assessment of early infarct in the anterior circulation (13, 15, 17–20, 22, 35). As shown in Table 1, all of the 10 recent RCTs used NIHSS scores as eligibility criteria (≥2, ≥5, ≥6, ≥8, ≥10, 8–29, and 10–25) (13–22). From these studies, there are insufficient data to determine whether there is an overall net benefit from EVT in patients with NIHSS score 2–5 (13, 15, 89). A NIHSS score ≥ 6 was the minimum used in 4 trials (14, 17, 20, 22), fulfilling the AHA's Level of A evidence. The other 4 trials used higher NIHSS score (≥ 8) (16, 18, 19, 21). Meta-analysis of the pooled data from the 5 RCTs published in 2015 showed strong efficacy of thrombectomy in patients with ASPECTS ≥ 6 (35). Therefore, NIHSS ≥6 and ASPECTS ≥ 6 from LVO are evidence-based cut-off values for timely decision-making for thrombectomy (90, 91).

**Figure 3:**
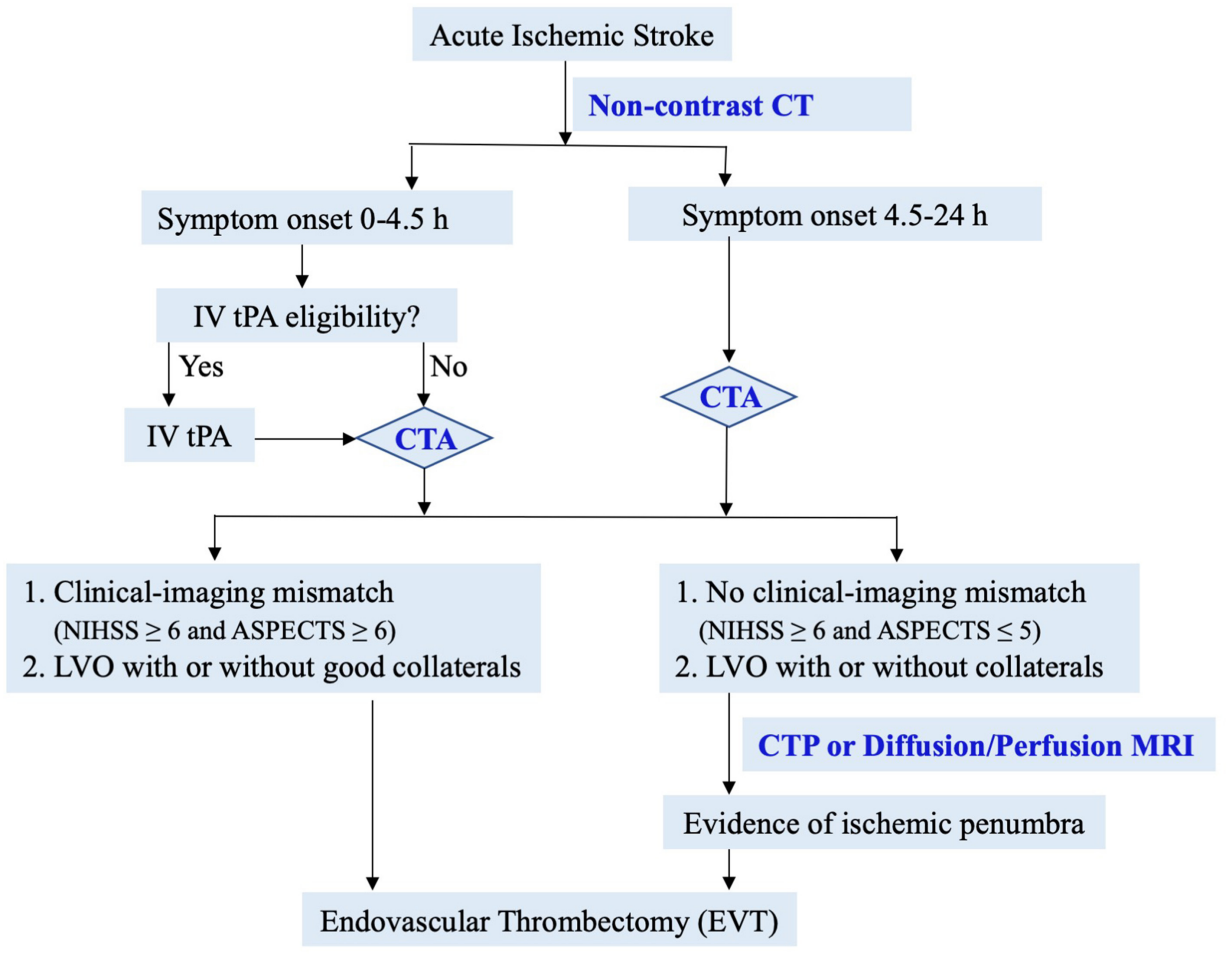
Proposed simple imaging guide for endovascular thrombectomy.

Based on data from recent landmark studies, we propose the following simple and efficient imaging guide for decision-making for EVT (Figure 3). In patients with suspected acute ischemic stroke, non-contrast CT is performed to assess IV tPA eligibility and ASPECTS score. CTA is then performed to evaluate LVO and collaterals. In patients with significant clinical-imaging mismatch (NIHSS ≥6 and ASPECTS ≥6) from LVO, EVT should be considered immediately per AHA guidelines (90, 91). In patients without clinical-imaging mismatch (NIHSS ≥6 and ASPECTS ≤ 5), advanced perfusion imaging is recommended to identify salvageable ischemic penumbra. This simple and efficient imaging protocol may lead to EVT for most eligible patients in the fastest onset-to-reperfusion time. Two recent studies have shown the safety and effectiveness of simplified imaging protocol in patients with wake-up or late presenting stroke (89, 92).

Of note, ASPECTS has low inter-rater reliability, especially in early time window (32, 33, 93). For patients with a high ASPECTS and a LVO on CTA, the ASPECTS-based guideline is an easy and fast protocol to safeguard earliest treatment. In cases with low ASPECTS ( ≤ 5) or uncertain eligibility, a multimodal imaging study should be used to rescue patients with salvageable ischemia.

## Conclusion

EVT is a proven therapy for appropriately selected patients with AIS from LVO up to 24 h of symptom onset (13–22). Although advanced perfusion imaging may better define infarct core and ischemic penumbra, they have a number of limitations for the real-world practice. A simple imaging protocol with non-contrast CT and CTA to identify clinical-imaging mismatch (NIHSS ≥6 and ASPECTS ≥6) from LVO may be the best guide for EVT in clinical practice. Advanced perfusion imaging is recommended in patients with large infarct core to identify additional candidates for the best possible care.

## Author Contributions

WY contributed to literature review, manuscript draft and final revision. W-JJ contributed to discussions of important intellectual contents and manuscript revision.

### Conflict of Interest Statement

WY is a scientific consultant at Stryker Neurovascular. The remaining author declares that the research was conducted in the absence of any commercial or financial relationships that could be construed as a potential conflict of interest.

## Abbreviations

AIS: acute ischemic stroke
ASPECTS: Alberta Stroke Program Early CT Score
CT: computed tomography
EVT: endovascular thrombectomy
IQR: interquartile range
IVT: intravenous thrombolysis
OTT: onset to treatment time
RCTs: randomized controlled trials
sICH: symptomatic intracranial hemorrhage
tPA: tissue plasminogen activator
DAWN: Diffusion Weighted Imaging (DWI) or Computerized Tomography Perfusion (CTP) Assessment with Clinical Mismatch in the Triage of Wake Up and Late Presenting Strokes Undergoing Neurointervention
DEFUSE 3: Endovascular Therapy Following Imaging Evaluation for Ischemic Stroke
ESCAPE: Endovascular Treatment for Small Core and Anterior Circulation Proximal Occlusion with Emphasis on Minimizing CT to Recanalization Times
EXTEND-IA: Extending the Time for Thrombolysis in Emergency Neurological Deficits—Intra-Arterial trial
IMS III: Interventional Management of Stroke III trial
MR CLEAN: Multicenter Randomized Clinical Trial of Endovascular Treatment for Acute Ischemic Stroke in the Netherlands
MR RESCUE: Mechanical Retrieval and Recanalization of Stroke Clots Using Embolectomy
REVASCAT: Randomized Trial of Revascularization with Solitaire FR Device vs.Best Medical Therapy in the Treatment of Acute Stroke Due to Anterior Circulation Large Vessel Occlusion Presenting Within 8 h of Symptom Onset
SWIFT PRIME: Solitaire with the Intention for Thrombectomy as Primary Endovascular Treatment trial
SYNTHESIS EXP: Intra-Arterial vs. Systemic Thrombolysis for Acute Ischemic Stroke (SYNTHESIS EXP) trial
THERAPY, The Randomized: Concurrent Controlled Trial to Assess the Penumbra System's Safety and Effectiveness in the Treatment of Acute Stroke.
